# Creative Work as Seen Through the ATHENA Competency Model

**DOI:** 10.3390/bs15111469

**Published:** 2025-10-29

**Authors:** Jérémy Lamri, Karin Valentini, Felipe Zamana, Todd Lubart

**Affiliations:** 1 Tomorrow Theory, 75002 Paris, France; karin.valentini@tomorrowtheory.com; 2 The Aix-Marseille Graduate School of Management, Aix-Marseille University, 13100 Aix-en-Provence, France; 3 Laboratoire de Psychologie et d’Ergonomie Appliquées (LaPEA), Université Paris Cité and Univ Gustave Eiffel, F-92100 Boulogne-Billancourt, France; mail@felipezamana.com (F.Z.); todd.lubart@u-paris.fr (T.L.)

**Keywords:** creativity, competency model, ATHENA framework, instructional design, AI-assisted learning

## Abstract

This article introduces the ATHENA competency model, a systemic framework designed to conceptualize and support the development of creativity and complex skills in professional and educational contexts. Creativity, increasingly seen as essential across sectors, requires the coordination of cognitive, motivational, emotional, social, and sensorimotor resources. ATHENA conceptualizes competencies as emergent, agentic behaviors, not static possessions, arising from the coordination of five dimensions: cognition, conation, knowledge, emotion, and sensorimotion. These are subdivided into 60 facets, each described across four progressive mastery levels, enabling fine-grained diagnosis and developmental roadmaps. To operationalize this framework, ATHENA includes three modules: Skills, which models the requirements of professional tasks; Profile, which analyzes learner populations and contextual constraints; and LEARN, a repertory of pedagogical activities linked to ATHENA facets. The article illustrates the system through two case studies of creative job activities—graphic design and workshop facilitation—demonstrating how ATHENA aligns abstract competencies with practical training interventions. The model bridges theoretical research in psychology, creativity, and education with instructional design. Future work aims to refine its applicability, scalability, and cross-cultural relevance.

## 1. Introduction

Creativity is consistently identified as one of the key skills for the 21st century. Defined as the production of ideas that are both novel and appropriate to a given context, it plays a pivotal role in solving complex problems and enabling innovation (Amabile, 1983; Sternberg & Lubart, 1995). Beyond the cultural and artistic sectors in which it has traditionally been celebrated, creativity is increasingly recognized as a transversal driver of economic growth and social adaptation (Florida, 2019; Artige & Lubart, 2020; UNCTAD, 2024). It is central to the development of new products, services, and processes, but also to the transformation of organizations and societies facing unprecedented challenges (Landry, 2012; Zamana, 2022).

The professional landscape illustrates this dual movement. On the one hand, creative industries—advertising, design, performing arts, film, video games, or publishing—constitute a growing share of economic activity (Howkins, 2013). On the other, a broad range of professions, from engineers and educators to managers, lawyers, and healthcare practitioners, now require creative capacities to adapt to rapid technological, economic, and ecological change. This diffusion has prompted renewed efforts to identify the cognitive, motivational, emotional, and social factors that support creative performance (Feist, 1998; George, 2007; Lubart, 1999; Sternberg, 2011). A substantial body of research highlights the multivariate nature of creativity, involving not only divergent thinking, associative richness, or mental flexibility, but also conative traits such as openness or risk tolerance, as well as motivational and emotional regulation processes (Guilford, 1956, 1983; Kasirer & Shnitzer-Meirovich, 2021; Thornhill-Miller et al., 2023).

Despite this growing consensus, the field lacks a unified and operational framework capable of capturing the systemic nature of competencies underlying creative work. Existing approaches often remain fragmented: cognitive models focus on intellectual abilities, personality research emphasizes traits, while educational and managerial frameworks tend to reduce competencies to narrow taxonomies of knowledge and skills (Tardif, 2006; Mulder, 2014). As a result, training systems and assessment tools are frequently misaligned with the situated and dynamic processes that actually sustain creativity in professional contexts (Eraut, 2004; Le Boterf, 2008).

To address this gap, we build on previous work conceptualizing skills as bundles of task-specific ingredients (Lubart et al., 2013; Lamri & Lubart, 2023). These studies suggest that any job can be understood as a constellation of tasks, each mobilizing a distinct configuration of abilities, traits, motivations, and styles. A multivariate profile of these resources enables not only prediction of task performance, but also the design of targeted training interventions. Extending this logic, we propose the ATHENA competency model, a systemic framework that reconceptualizes competencies not as stable, isolated entities, but as emergent behaviors resulting from the agentic coordination of multiple internal resources.

ATHENA articulates five fundamental dimensions—cognition, conation, knowledge, emotion, and sensorimotor activity—into a detailed structure of 12 subdimensions for a total of 60 facets, with each of the facets described across four progressive levels of mastery. This architecture is designed to provide a “new grammar” for human competencies: precise enough to analyze the resources mobilized in a given professional situation, yet flexible enough to inform instructional design and adaptive training. By integrating insights from cognitive science, personality psychology, motivational theory, emotional intelligence, and embodied cognition, ATHENA aims to overcome the reductive separation of “hard” and “soft” skills, offering instead a holistic, integrative approach.

To clarify the terminology used in this framework, each dimension refers to one of the five broad functional domains that constitute the ATHENA framework—Cognition, Conation, Knowledge, Emotion, and Sensorimotion. Within each dimension, a sub-dimension groups conceptually related aspects of functioning; for instance, within Conation, the sub-dimensions Motivation, Volition, and Adaptability capture distinct yet complementary processes of goal-directed action. The most granular units of analysis, called facets, represent specific internal resources or behavioral capacities (e.g., divergent thinking, self-regulation, emotional perception). In ATHENA, the dynamic interaction among these facets within and across dimensions forms the basis of competent performance.

The objective of this article is threefold. First, to present the theoretical foundations of the ATHENA model and situate it within the broader literature on intelligence, creativity, and professional skills. Second, to illustrate its operationalization through the design of a digital educational tool composed of three modules—Skills, Profile, and Learn—that support instructional designers in modeling professional tasks, characterizing learner populations, and orchestrating targeted pedagogical sequences. Third, to demonstrate the model’s applicability through two case studies (graphic design and workshop facilitation), with a detailed example of pedagogical scenarios for the development of divergent thinking.

In doing so, we aim to contribute to the development of a systemic, evidence-based framework for creativity and complex skill development, bridging theoretical research and practical applications in education and professional training. The ambition is not to offer a universal model, but rather an evolving structure that can guide empirical validation, adapt to diverse professional contexts, and integrate technological advances in AI-assisted learning while remaining anchored in a human-centered epistemology of skills.

In the following section, we detail the theoretical underpinnings of ATHENA, showing how the model emerges from and extends existing work in psychology, education, and the science of creativity, before turning to its operational implementation.

## 2. Theoretical Foundations of the ATHENA Model: Towards a New Grammar of Human Skills

Despite significant advances in educational and psychological sciences, competencies continue to be modeled by many institutions and organizations through frameworks that are conceptually fragile or empirically outdated. For example, the tripartite distinction between knowledge, know-how, and interpersonal skills remains widely used in vocational and educational systems. However, this taxonomy lacks a solid empirical foundation and reduces the dynamic complexity of learning processes to operational but oversimplified categories (Tardif, 2006; Mulder, 2014).

This situation reflects a deeper epistemological problem: the absence of consensus on the very definition and scope of competencies. As a result, competencies are frequently instrumentalized for purposes of certification or managerial control, rather than grounded in the real psychological processes of cognition, conation, or emotion activated in context (Mohammed & Ozdamli, 2024; Le Boterf, 2008; Eraut, 2004). This structural gap between prescribed models and the situated reality of skill deployment has well-documented consequences: assessments that poorly predict performance, training systems that fail to address developmental needs, and career guidance tools that rest on biased or unclear representations of human potential (Jung et al., 2022; Liang et al., 2023; Zhou et al., 2024; Perrenoud, 2004). Similar critiques have been advanced in the French-speaking literature on formative assessment (Allal, 2002) and in interpretative approaches to competence at work (Sandberg, 2000).

In response to these theoretical and operational limitations, the ATHENA model introduces a conceptual shift. Table 1 shows a comparison between major skills frameworks and ATHENA. Rather than defining competencies as categorical, stable entities objectifiable in isolation, ATHENA conceives them as emergent, situated behaviors that arise from the synchronized mobilization of diverse internal resources. In this perspective, competencies are not “things” one possesses but agentic activations—the capacity of an individual to intentionally coordinate multiple cognitive, conative, affective, knowledge-based, and sensorimotor resources in response to contextual demands. The analogy with bodily movement is instructive: just as movement never results from the isolated action of a single muscle, competent performance cannot be attributed to a single dimension or facet. It is the outcome of systemic articulation between intentions, representations, regulatory processes, and contextual requirements.

The ATHENA model builds on and extends existing multivariate approaches to intelligence and creativity (Amabile, 1983; Sternberg & Lubart, 1995; Lubart et al., 2013; Lubart, 2017; Lamri & Lubart, 2023). It articulates five foundational dimensions, each structured into categories and facets that render them operational for analysis and instructional design:

**Cognition** encompasses information processing, reasoning, memory, attention, and innovation. It integrates contributions from differential psychology (Cattell, 1987) and models of executive functioning emphasizing inhibition, shifting, and updating (Miyake et al., 2000). Within ATHENA, this dimension includes reasoning facets such as inductive reasoning, deductive reasoning, and abstract reasoning; memory facets like short-term memory, long-term memory, and working memory; attentional resources including sustained attention, selective attention, and divided attention; as well as creative–innovative resources such as divergent thinking, cognitive flexibility, and conceptual synthesis.

- **Conation** refers to volition, motivation, and perseverance, and is informed by theories of motivation and personality (Deci & Ryan, 2000; Kuhl, 1985). In ATHENA, it is broken down into categories such as motivation (intrinsic motivation, extrinsic motivation, self-efficacy), volition (self-regulation, perseverance, time management), proactivity (initiative, decision-making, problem solving), and adaptability (behavioral flexibility, uncertainty management, lifelong learning). Together, these facets capture the dynamic capacity of individuals to sustain, orient, and adapt their actions in uncertain or challenging contexts.
- **Knowledge** is conceived not simply as the possession of facts but as structured and mobilizable resources. Following expertise research (Chi et al., 1988), ATHENA distinguishes between declarative knowledge (factual knowledge, concepts, principles), procedural knowledge (technical expertise, algorithms, heuristics), metacognitive knowledge (learning strategies, self-awareness, cognitive self-regulation), and contextual knowledge (knowledge transfer, systems thinking, cultural intelligence, awareness of trends). This dimension emphasizes that adaptive expertise lies in both depth and transferability of knowledge.
- **Emotion** refers to the awareness, regulation, and productive use of affective states in learning and performance, grounded in emotional intelligence (Mayer & Salovey, 1997) and regulation models (Gross, 2002). ATHENA details emotional competence (emotional perception, emotional regulation, adaptive use of emotions), resilience (stress adaptation, optimism, post-traumatic growth), and empathy (emotional recognition, perspective taking, compassion, social intelligence). These facets highlight how creative and professional performance is inseparable from the affective resources that sustain collaboration, motivation, and adaptation.
- **Sensorimotion** emphasizes the embodied and perceptual dimensions of competence, in line with embodied cognition (Varela et al., 1991) and motor learning research (Schmidt & Lee, 2011). ATHENA distinguishes perceptual acuities (visual acuity, auditory acuity, proprioception), coordination (hand–eye coordination, balance, fine dexterity), motor performance (speed, precision, endurance, automatization), and sensorimotor integration (functional synesthesia, sensorimotor adaptation, sensory compensation). These resources remind us that competence is not only cognitive or emotional but always grounded in bodily engagement with the environment.

Unlike existing models that separately address cognitive, emotional, motivational, or knowledge-based components of performance, ATHENA integrates these five domains within a single, agentic framework. While its overall structure may appear reminiscent of prior approaches such as KSAOs (Knowledge, Skills, Abilities, and Other characteristics; Fleishman & Reilly, 1992), Gardner (1983)’s Multiple Intelligences, or the Four-C model of creativity (Kaufman & Beghetto, 2009), ATHENA differs on three essential points.

First, it conceptualizes competencies as emergent behaviors rather than stable traits, explicitly linking performance to the agentic coordination of multiple internal resources. Second, it operationalizes this principle through an unprecedented level of granularity, proposing 60 facets distributed across five dimensions and articulated through four progressive mastery levels, providing a psychometric and developmental roadmap absent from earlier frameworks. Third, ATHENA embeds this ontology within a technologically assisted pedagogical system that connects each facet to AI-supported instructional methods, thereby extending traditional competency models into the domain of adaptive learning design.

By integrating these five dimensions, ATHENA resonates with the holistic and systemic orientation of socio-cultural and activity-theoretical perspectives, which stress the integrative, adaptive, and situated nature of professional development (Engeström, 2001).

To render this architecture actionable, the ATHENA model identifies 60 facets distributed across the five dimensions, as stated earlier. Each facet captures a specific internal resource—such as working memory, divergent thinking, uncertainty management, perseverance, emotional regulation, or hand–eye coordination—that may be differentially mobilized depending on the demands of a given task. This fine granularity allows for both diagnostic precision (e.g., identifying bottlenecks in performance dynamics) and targeted developmental interventions.

Furthermore, each facet is described across four progressive levels of mastery—novice, intermediate, advanced, and expert. These levels reflect not only the degree of skill mobilization but also the stability, autonomy, and transferability of performance. For example, novice-level self-regulation corresponds to emergent and highly contextualized mobilization, while expert-level self-regulation reflects fluid and adaptive regulation across varied contexts, often with automatic or reflexive activation. This graded structure provides a roadmap for learning trajectories and ensures alignment between assessment, instructional design, and developmental goals.

At the heart of ATHENA lies the principle of agency (Wiśniewska & Karwowski, 2025). Competence is defined not merely as a sum of abilities or traits, but as the intentional activation of resources toward a meaningful outcome. This aligns with contemporary views of creativity and intelligence as dynamic, distributed processes rather than static traits. Recent theoretical contributions—such as the consensual definition of creativity (Plucker et al., 2004), the Four C model distinguishing different levels of creative expression (Kaufman & Beghetto, 2009), and the Dual Pathway model highlighting flexibility and persistence as complementary routes to creativity (De Dreu et al., 2012)—all converge on the notion that adaptive performance requires a coordinated interplay of multiple psychological processes (Ippoliti et al., 2024; Mekern et al., 2019; Pinkow, 2023).

Neuroscientific evidence further supports this view. Creativity is increasingly understood as the product of dynamic coupling between large-scale brain networks, especially the default mode, executive control, and salience networks (Khalil et al., 2019; Beaty et al., 2016; Benedek et al., 2014). These findings highlight the need to move beyond single-factor models, toward frameworks capable of capturing multivariate, flexible, and context-sensitive activation—precisely the orientation of ATHENA.

ATHENA also responds to gaps in the literature on training for creativity and complex skills. Meta-analyses demonstrate that creativity training is most effective when it targets multiple components, combines divergent and convergent processes, and provides opportunities for transfer across contexts (Oh & Pyo, 2023; Papachristopoulos et al., 2023; Scott et al., 2004; Ma, 2006). This evidence underlines the necessity of systemic frameworks that can guide the design of such interventions. ATHENA’s ontology of facets and mastery levels provides exactly this scaffolding: a structured yet flexible grammar for designing instructional strategies that align learner resources with task requirements.

Taken together, the theoretical foundations of ATHENA advance the field in three ways. First, they offer a systemic ontology that transcends the hard/soft skill divide and situates competence as emergent, agentic activation. Second, they provide granularity (60 facets × 4 levels) that allows for detailed diagnosis and instructional alignment. Third, they build a bridge between theory and practice, integrating established psychological research with a framework that can be directly operationalized in educational and professional contexts.

ATHENA is thus conceived not as a fixed reference system but as a mediation structure: an evolving, integrative, and human-centered framework designed to be refined through empirical validation and enriched by technological advances in AI-assisted learning.

## 3. From Theory to Platform: ATHENA Educational Design in Action

The transition from the theoretical ATHENA model to its operational implementation was achieved through the design of a digital educational engineering tool, based on the scientific literature, and powered by several generative AI models.

The ATHENA tool consists of three interdependent modules: Skills, Profile, and Learn. These three modules were designed to model, contextualize, and orchestrate targeted learning paths, particularly in contexts of professional transformation or the acquisition of complex skills. The whole is based on a declarative, structured, and interpretative approach, in which the educational designer plays a central role in mediation and validation. Each module is described below in terms of its own logic, its distinctive contributions, and its integration into the general ATHENA ecosystem.

The Skills module is the main entry point into the modeling logic. Its function is to objectify the specific requirements of a given professional activity or job through a multidimensional grid based on the 60 facets of the ATHENA model. This operation is based on declarative work carried out by the educational designer, who completes an activity analysis form. The latter includes the functional description of the targeted task as well as the selection, within the ATHENA nomenclature, of the relevant facets.

To ensure consistency, interoperability, and semantic relevance in the operational use of the ATHENA model, conceptual standardization work was necessary prior to modeling. More specifically, it was necessary to clarify what was meant by “competency” in the operational sense of the term in order to allow for a clear and stable structuring of user inputs in the Skills module. To achieve this, the ATHENA model is based on the European nomenclature ESCO (European Skills, Competences, Qualifications and Occupations), which provides a unified taxonomy of transversal and professional skills used in many employment and training systems across Europe (European Commission, 2023).

When a user wishes to model a skill with the Skills module, the system automatically suggests a correspondence with the ESCO database, selecting the entry semantically closest to the entered formulation. This operation is ensured by a natural language processing function, combining lexical recognition and contextual weighting. In cases where no explicit correspondence can be established, a conversational assistant based on a generative language model is used to assist the user in reformulating their initial intention. This assisted dialogue not only refines the designation of the target skill but also makes the user aware of the conceptual distinctions underlying their request. This hybrid process, combining ontological standardization and dialogical interaction, thus guarantees increased robustness of skills profiling, a prerequisite for the reliability of subsequent educational recommendations.

For each of the facets selected, the pedagogical designer is required to qualify the expected frequency of use in the professional situation (for example, whether the requirement is permanent, one-off or occasional), the level of intensity required to perform satisfactorily, as well as the systemic role of the facet in the entire activity (i.e., its essential, secondary or complementary nature). These three parameters make it possible to generate a systemic graphic representation of the activity requirements. The objective is not to produce a prescriptive reference framework, but to provide an analytical basis to inform situated pedagogical choices.

The interest of the Skills module lies in its ability to transform an often implicit and fragmentary description of professional requirements into an explicit, structured, and usable structure. This modeling makes it possible to read skills across the board, to compare related or contrasting activities, and to anticipate areas of difficulty or over-solicitation in the associated training systems.

The Profile module completes the analysis by providing a qualitative characterization of the learner population concerned by the educational program. The module is based on a global declarative assessment, formulated by the educational designer. The designer informs a set of structured fields aimed at qualifying the average level of experience of the cohort on the targeted activity, the resources perceived as present or absent in the group, integrate certain pedagogical preferences that may reflect the organization’s culture (such as preference for cooperative learning, playful approaches, or other distinctive instructional styles, as well as anticipated emotional or motivational dynamics (for example, adherence to the training project, resistance to change or initial confidence).

The data thus provided do not allow individual psychological profiles to be inferred, but they do help establish a hypothesis of consistency between the requirements modeled by the Skills module and the resources available in the group of people to be trained. Cross-referencing these two sources of information generates an aggregated profile of the cohort, presented in the form of a comparative mapping of alignment areas (where requirements and resources are compatible) and areas of tension or deficiency (where a gap is identified between what is required and what is estimated to be available). The Profile module thus provides decision-making support to anticipate the pedagogical efforts of compensation or reinforcement to be implemented, without claiming to be an exhaustive or normative evaluation of the trained population.

The third module, Learn, constitutes ATHENA’s pedagogical scenario interface. It is based on a database of more than two hundred identified pedagogical techniques and methods, each documented according to its main effects on the facets of the ATHENA model, its deployment formats (face-to-face, distance, synchronous, asynchronous), its conditions of applicability (duration, level of autonomy required, logistical framework), and the desired effects in terms of cognition, engagement, cooperation or transfer. These methods are derived from a systematic review of the literature in instructional engineering, learning sciences, and experiential instructional design.

Based on the cross-referencing of activity requirements (Skills module) and cohort characteristics (Profile module), the Learn module proposes a set of educational sequences deemed relevant, organized according to an algorithmic relevance ranking. Each proposal is accompanied by a descriptive sheet specifying its educational intention, the potentially stimulated facets, the implementation constraints, and the possible variants.

It should be emphasized that these suggestions are intended as recommendations from the ATHENA system that need to be reviewed, adapted, and validated by the instructional designer. The latter can accept them as they are, adjust them, combine them, or reject them. The human being thus remains at the heart of the decision-making process, in a logic of assistance and not substitution.

It is important to note that although the ATHENA model was designed to eventually integrate recursive logic based on usage traces and feedback (learning analytics), no algorithmic learning loop is currently deployed in the operational modules. The proposed structure, therefore, remains declarative, hypothetico-deductive, and assisted, without adaptive automation in real time. In future developments, ATHENA Learn could be continuously enriched by a community of educators or experts in the relevant facets. This approach could enable the creation of a sort of “Wikipedia of pedagogy”, relying both on the relevance of the pedagogical solution to the targeted facets and on the creativity and originality of the proposal.

## 4. Putting ATHENA in Action: Two Examples of Relating to Creative Thinking

In this section, we present two instructional-design demonstrations that illustrate how the ATHENA system can be applied to conceptualize creative skills within complex professional tasks. These cases should be viewed as pilot illustrations rather than controlled empirical studies, following a design-based research logic (Reeves & McKenney, 2019). Their purpose is to exemplify how the model translates theoretical constructs into operational pedagogical pathways, not to measure training effectiveness or comparative performance outcomes.

Future research will build on these preliminary demonstrations through systematic validation protocols, including pre-/post-test evaluations, expert-rating comparisons, and learner-feedback analyses, to assess the pedagogical impact of ATHENA-based designs.

### 4.1. Example 1: “Graphic Design” Conceived Through Athena Skills

Graphic design is seen as a creative task involved in a wide range of creative industries. Based on ESCO, Graphic design involves creating and structuring relevant, effective, and aesthetically pleasing visual content with the specific goal of clearly communicating a message, idea, concept, or visual identity. It involves mastering technical digital tools (graphic design software such as Photoshop, Illustrator, or InDesign), manufacturing processes (such as printing in different materials and surfaces), fundamental composition principles (typography, colors, layout, visual hierarchy), and analytical skills to understand the needs of a client or project. In concrete terms, this skill allows for the production of a variety of media such as logos, posters, and printed or digital documents, responding creatively, coherently, and impactfully to the expectations of target audiences.

Ranking by decreasing importance of the ATHENA facets (for the 12 most solicited facets among the 60 facets of the model), we obtain a set of facets, each connected with one of the five ATHENA dimensions, and for each facet there is the level of mastery needed:

#### 4.1.1. Technicality (Knowledge Dimension—Expert Level)

Technical expertise refers to the specific know-how and practical mastery of digital graphic design tools such as Photoshop, Illustrator, or InDesign, as well as know-how in materials (e.g., different types of paper, ink, textiles, etc.) and the use of manual tools to specific ends, such as brushes for painting, pencil and pens for drawing, and so on. It is the essential foundation of graphic design, enabling the precise and professional execution of any graphic design project.

#### 4.1.2. Divergent Thinking (Cognition Dimension—Expert Level)

This ability to generate innovative, original, and creative ideas is essential for designing visuals that stand out. It fuels visual innovation and helps meet the diverse needs of clients while surprising and captivating the audience. A well-known creative process used by designers is the Design Thinking process.

#### 4.1.3. Hand–Eye Coordination (Sensory Motion Dimension—Expert Level)

Hand–eye coordination provides the physical mastery necessary for precision in graphic design tasks such as drawing, meticulous tracing, or fine-tuning the details. It guarantees impeccable technical quality.

#### 4.1.4. Sustained Attention (Cognition Dimension—Expert Level)

This skill allows the designer to remain focused on precise details for a long time, avoiding errors and ensuring visual consistency in complex and detailed graphic projects, such as being attentive to color matching, objects’ shape and size, semiotics rules, grid composition, etc.

#### 4.1.5. Facts—Declarative Knowledge (Knowledge Dimension—Expert Level)

This knowledge of the fundamental rules of typography, color, layout, and visual hierarchy provides a solid foundation upon which all quality graphic design is based. Mastering this knowledge allows for professional, consistent, and visually pleasing creations.

#### 4.1.6. Behavioral Flexibility (Conation Dimension—Expert Level)

The ability to quickly adapt to changing contexts or new demands is essential when faced with frequent adjustments to graphic design projects. It allows for easy integration of client feedback or brief modifications, while maintaining an effective creative approach.

#### 4.1.7. Inductive Reasoning (Cognition Dimension—Advanced Level)

This skill allows general graphic principles to be drawn from specific examples, facilitating the rapid identification of trends, target audience expectations, and sponsor intentions.

#### 4.1.8. Knowledge Transfer (Knowledge Dimension—Advanced Level)

Knowledge transfer facilitates the adaptation of graphic skills and knowledge from one context to another, allowing the designer to move easily from a digital medium to a printed medium, or from a visual identity project to a poster or a website.

#### 4.1.9. Intrinsic Motivation (Conation Dimension—Advanced Level)

Intrinsic motivation fuels creative energy and supports the designer’s long-term commitment to their projects, enabling them to overcome difficulties and maintain a high level of creative and technical performance.

#### 4.1.10. Self-Regulation (Conation Dimension—Advanced Level)

Self-regulation ensures good personal management of time, resources, and energy, essential to the success of graphic projects that require rigor, method, and respect for deadlines.

#### 4.1.11. Emotional Perception (Emotion Dimension—Advanced Level)

Emotional perception allows us to precisely grasp the expected emotional impact of a design on its target audience, reinforcing the communication effectiveness of the visual and facilitating the creation of graphic media capable of reaching and influencing the target audience.

#### 4.1.12. Short-Term Memory (Cognition Dimension—Intermediate Level)

Finally, short-term memory helps to manage multiple pieces of graphic information simultaneously, facilitating the rapid manipulation of ideas and visual elements during the creative and technical process.

### 4.2. Example 2: “Workshop Facilitation” Skills Conceived Through Athena Skills

Workshop facilitation skills are essential in creative problem-solving sessions. Based on ESCO, the definition of Workshop facilitation involves organizing, structuring, and leading collaborative sessions to effectively achieve a specific objective (creation, innovation, problem-solving, decision-making, collective learning, etc.). This skill involves knowing how to design a coherent sequence of activities, manage group dynamics, stimulate constructive exchanges, and ensure the active and balanced participation of everyone. A good facilitator masters specific facilitation methods (brainstorming, design thinking, icebreakers, educational games, etc.), time and collective energy management techniques, and active listening to adapt to changing interactions to maximize the impact of the workshop.

The 12 most relevant ATHENA facets, ranked by decreasing importance, are distributed across the five ATHENA dimensions, each with its corresponding mastery level.

#### 4.2.1. Behavioral Flexibility (Conation Dimension—Expert Level)

Behavioral flexibility allows us to quickly and effectively adjust behavior, methods, and approaches based on how the group and the process evolve. This is essential for adapting the animation in real time to the specific needs and reactions of the participants.

#### 4.2.2. Self-Regulation (Conation Dimension—Expert Level)

Self-regulation ensures excellent personal control over time, energy, and emotions during the workshop. It is essential for effectively structuring activities, respecting planned timing, and maintaining a positive and fluid dynamic.

#### 4.2.3. Knowledge Transfer (Knowledge Dimension—Expert Level)

Knowledge transfer ensures the relevant and adapted use of various facilitation methods (brainstorming, icebreakers, design thinking) according to the specific objectives of each workshop. This skill allows for great methodological flexibility in varied contexts.

#### 4.2.4. Emotional Perception (Emotion Dimension—Expert Level)

Emotional perception allows for a precise understanding of participants’ emotions and their impact on group dynamics. It is a key skill for fostering a climate conducive to constructive exchange, effectively managing potential tensions, and ensuring the quality of interactions.

#### 4.2.5. Sustained Attention (Cognition Dimension—Advanced Level)

Sustained attention helps maintain constant focus on the precise sequence of activities, while remaining vigilant for the unexpected. It is essential for managing delicate moments and ensuring the efficient and smooth running of the workshop.

#### 4.2.6. Divergent Thinking (Cognition Dimension—Advanced Level)

This ability to generate a large number of creative and innovative ideas is particularly useful for designing original, stimulating activities adapted to the workshop objectives, thus enabling better engagement of participants.

#### 4.2.7. Intrinsic Motivation (Conation Dimension—Advanced Level)

Intrinsic motivation fosters a positive and contagious energy that strengthens group engagement. It allows the facilitator to remain dynamic and enthusiastic throughout the workshop, thus facilitating the active and lasting involvement of participants.

#### 4.2.8. Emotional Recognition (Emotion Dimension—Advanced Level)

Emotional recognition, which complements emotional perception, allows participants to identify and respond appropriately to their emotions. This skill is essential for creating an environment of trust and mutual respect that fosters authentic exchanges.

#### 4.2.9. Initiative (Conation Dimension—Advanced Level)

The initiative allows for anticipating the group’s needs, actively promoting participation, and stimulating discussion. It directly contributes to the proactive facilitation of the workshop and ensures balanced involvement of all participants.

#### 4.2.10. Adaptation to Stress (Emotion Dimension—Advanced Level)

Stress adaptation is important for effectively managing difficult or complex situations (conflicts, resistance, unforeseen events). It allows the facilitator to remain calm, effective, and maintain a positive atmosphere despite potential tensions.

#### 4.2.11. Short-Term Memory (Cognition Dimension—Intermediate Level)

Short-term memory helps you efficiently and simultaneously manage the multiple pieces of information needed during the workshop: participant contributions, points discussed, and interim objectives. It thus facilitates the flow of the workshop and the coherence of the discussions.

#### 4.2.12. Hand–Eye Coordination (Sensory Motion Dimension—Intermediate Level)

Finally, hand–eye coordination ensures the fluid use of animation tools (interactive boards, visual aids, digital tools), thus facilitating dynamic and concrete animation, visually clear and stimulating for participants.

## 5. ATHENA Profile

This part of the ATHENA system provides the foundation for the contextualization of learning design. It allows instructional designers to define the pedagogical environment within which skills development will take place, thereby informing the selection of sequences in the ATHENA Learn module. In other words, ATHENA Profile serves as a bridge between the learner cohort’s constraints and resources, and the system’s capacity to recommend pedagogical interventions that are not only relevant, but also feasible and sustainable.

The purpose of ATHENA Profile is to specify the learners’ collective profile across a set of parameters that have been shown to strongly influence the effectiveness of instructional design:

(a) Language (e.g., whether training is delivered in a single language, in multiple languages simultaneously, or requires translation and subtitling to ensure accessibility);
(b) Temporal availability (e.g., whether learners can dedicate one day, one week, or several months, and how this time is structured across their schedules);
(c) Temporal sequence (e.g., whether learning should be concentrated in intensive sessions or spread out in a distributed learning sequence such as weekly workshops, reflecting evidence that spacing effects enhance long-term retention);
(d) Physical or digital availability (e.g., fully in-person, fully online, or hybrid learning contexts, each of which brings distinct pedagogical opportunities and logistical constraints);
(e) Social setting (e.g., training delivered in individual mode, within small peer groups, or to large cohorts, with implications for collaboration, peer learning, and feedback dynamics).
(f) The trigger for the training initiative and the organization’s training culture: for example, is the training initiated by employee requests, or is it driven by top management? Is it part of a strategic transformation effort, a regulatory requirement, or another type of organizational priority? Are managers supportive and actively involved, or distant from the training process?

These parameters have important implications for the design of the learning experience, such as the structure and length of onboarding or inclusion phases, the presence or absence of organizational leadership within the program, and the extent to which pedagogical design must emphasize motivational engagement of the participants (Carré, 2020).

By formalizing these parameters, ATHENA Profile generates a structured representation of learner and contextual constraints. This representation ensures that the subsequent pedagogical recommendations are not designed in the abstract, but are grounded in the realities of the learning situation. It also enables the system to balance hard constraints (such as mandatory online delivery for remote learners) with soft preferences (such as a cohort’s interest in small-group interaction), allowing designers to prioritize pedagogical efficiency while maintaining adaptability.

As a hypothetical example, imagine a cohort of learners receiving training to enhance their competency in graphic design. The cohort is multilingual, composed of master-level designers, and available for a three-month training program consisting of 20 lessons. Sessions must be delivered online in an individualized format. Under these conditions, the ATHENA Profile directs ATHENA Learn to prioritize individualized, artifact-based learning activities that can accommodate multilingual delivery (e.g., subtitled video tutorials, self-paced design challenges, or automated feedback mechanisms).

In a contrasting scenario, consider a workshop facilitation program aimed at a cohort of team managers. Here, the learners are all English speakers, available for a two-week intensive residential setting, and expected to learn collaboratively. In this case, ATHENA Profile guides ATHENA Learn toward pedagogical activities that leverage the in-person format and the group dynamic, such as role-play, peer observation, and real-time facilitation exercises, while ensuring that assessment and feedback mechanisms are aligned with the intensive temporal structure.

In both examples, ATHENA Profile demonstrates its role as an adaptive interface: by integrating information about learners’ linguistic, temporal, spatial, and social contexts, it enables ATHENA Learn to produce recommendations that are both pedagogically relevant and logistically viable. This ensures that the development of a given skill at a specific target level is not only theoretically sound, but also practically achievable within the learners’ real-world constraints.

## 6. ATHENA Learn—Selection of Pedagogical Activities

In the third part of the ATHENA system, ATHENA Learn, functions as a repertory of pedagogical activities designed to generate appropriate learning sequences according to the targeted learning goals and the learner’s level of expertise.

The construction of the ATHENA Learn database followed a three-step process. First, a literature-based inventory was compiled, comprising nearly 200 teaching techniques. These techniques, defined as sets of gestures, actions, devices, or strategies employed by instructors to facilitate learning, engage learners, and achieve educational objectives, were drawn from a variety of established pedagogical approaches, including transformational learning, vicarious learning, double-loop learning, and cooperative learning. Illustrative examples include hackathons, mind mapping, world cafés, forum theatre, design fiction, logbooks, shadowing, and AI-assisted learning.

Then, generative AI was used to assist expert pedagogical designers in associating each teaching technique to determine the techniques best suited to each ATHENA dimension (cognition, conation, knowledge, emotion, sensorimotor) according to the criteria presented in Table 2.

In the second step, AI-assisted selection processes were applied to determine the most suitable techniques for each level of mastery within the facets of each dimension. Pedagogical experts then validated and refined these selections based on the criteria outlined, as shown in Table 3.

In the third step, an AI-assisted selection of four to six teaching techniques were retained for each level of mastery, subsequently translated into examples of synchronous and asynchronous teaching activities, where both modalities were relevant.

We illustrate the result of this approach to pedagogical design in the example provided in Table 4. This example focuses on divergent thinking, which is included in both the skill set for graphic design and workshop facilitation, albeit at different levels of mastery: an expert level (Level 4) for graphic design, and an advanced level (Level 3) for workshop facilitation.

Table 4 shows an example of pedagogical activities relevant to develop divergent thinking, organized by expertise level.

It is important to note that the determination of the activities presented is carried out with the help of AI (ChatGPT-4o and Claude 3.1 in particular), but all the activities have been subjectively reread/enriched by human experts. A study is currently being conducted with a sample of around fifty instructional designers from 3 different organizations to evaluate the relevance of the proposed activities and enrich the proposals.

The list of activities proposed for each level of mastery of each facet does not claim to be exhaustive. It can and should be enriched with other examples. Ultimately, users of the ATHENA platform, through their choices of contextualization of the cohort and given their pedagogical expertise, will enable the selection of the activity most suited to each need. ATHENA falls within a logic of combining theoretical contributions (pedagogical approaches and techniques), the use of AI, and the critical eye of the human expert in skills development. These examples of teaching activities are not the final output produced by ATHENA Learn. They represent, in a way, the “raw data” provided to the AI. The final result communicated to the user integrates the contextualization elements from ATHENA Learn, thereby producing a more tailored and refined training program.

To return to our examples, for training to reach the expert level of divergent thinking in the graphic design task, conceptual hybridization or cognitive disruption strategy would be the most relevant, given that verbal domain pedagogical experiences are less domain-related and that the profile for learners as specified earlier is distance, individualized training. In contrast, for the second example, level 3 pedagogical experiences are aligned with the learning goal. In addition, the profile of learners (in person, interaction-oriented) favors the choice of either reflexive matrices or random stimuli. The final decision on the best pedagogical activity is, of course, made by the pedagogical designer who can take further contextual information on the task, the learners, or outside resources available and feasibility into account.

## 7. Discussion

The present study introduced the ATHENA competency model as a systemic framework to conceptualize, model, and support the development of creative work. By articulating five dimensions—cognition, conation, knowledge, emotion, and sensorimotion—into sixty operational facets distributed across progressive levels of mastery, ATHENA aims to provide both a conceptual “grammar” of human competencies and a practical foundation for instructional design. The empirical illustrations in the domains of graphic design and workshop facilitation highlight the model’s ability to capture the multivariate and context-dependent nature of creative performance, while offering concrete pathways for pedagogical intervention.

### 7.1. Theoretical Contributions

From a theoretical standpoint, ATHENA contributes to bridging the persistent fragmentation of competency models in psychology and education. Whereas traditional frameworks have tended to isolate cognition, personality traits, or knowledge domains, ATHENA emphasizes the agentic coordination of multiple internal resources. This resonates with multivariate approaches to creativity (Amabile, 1983; Sternberg & Lubart, 1995; Plucker et al., 2004) and with neuroscientific evidence highlighting the dynamic interplay of large-scale brain networks in creative cognition (Beaty et al., 2016). The model thus advances the field by situating competencies as emergent, dynamic, and distributed phenomena rather than as static possessions.

Another theoretical contribution lies in the explicit integration of embodied and emotional dimensions. While cognition and knowledge have long dominated models of skill acquisition, ATHENA incorporates emotion and sensorimotor resources as equally central. This inclusion echoes the embodied cognition paradigm (Varela et al., 1991) and the growing recognition of emotional intelligence in creative and professional performance (Mayer & Salovey, 1997). By doing so, ATHENA moves toward a holistic ontology that avoids the reductive “hard vs. soft skills” dichotomy.

### 7.2. Practical Implications for Instructional Design

In operational terms, the ATHENA platform illustrates how the model can be applied to pedagogical engineering. By combining declarative modeling (Skills module), contextual profiling (Profile module), and a repertory of teaching methods (Learn module), it enables instructional designers to align activity requirements with learner resources in a systematic manner. This alignment provides a structured pathway for designing training programs that target not only cognitive processes but also motivational, emotional, and embodied facets of creative work.

The two case studies demonstrate this potential. For graphic design, the model highlights the interdependence of technical expertise, divergent thinking, hand–eye coordination, and emotional perception. For workshop facilitation, it emphasizes behavioral flexibility, emotional regulation, and collaborative knowledge transfer. In both cases, ATHENA translates abstract competencies into operational facets that can be addressed through specific pedagogical activities, thereby reducing the frequent gap between competency frameworks and actual training practices (Eraut, 2004).

### 7.3. Benefits of the ATHENA Approach

Several benefits can be emphasized. First, the model provides granularity: by distinguishing sixty facets across four mastery levels, it allows for fine-grained diagnosis and developmental roadmaps. Second, it supports transferability across domains: although applied here to creative professions, the same structure can be mobilized for other complex skill sets, from engineering to healthcare. Third, its integration with ESCO facilitates interoperability with international competency frameworks, enhancing its potential adoption in diverse educational and professional contexts. Finally, the design of ATHENA as a human-centered system, where AI provides assistance but instructional designers retain agency, ensures that technological mediation enhances rather than replaces expert judgment. An additional benefit of ATHENA could be that ATHENA may stimulate the learning designer’s creativity by suggesting techniques they might not have initially considered, and by inviting them to combine these with their own experience.

### 7.4. Limitations and Challenges

Despite these contributions, several limitations must be acknowledged. First, the model remains largely conceptual and its empirical validation is ongoing. While the proposed facets are grounded in established psychological constructs, their precise delineation, interrelations, and developmental trajectories require systematic testing. Longitudinal studies and experimental interventions will be necessary to assess whether ATHENA-based training produces measurable improvements in creative performance. The two case studies presented in this paper are illustrative applications of the model’s design logic rather than empirical validations. Their function is conceptual demonstration; therefore, outcome data or control comparisons were intentionally not included at this stage.

Second, the reliance on declarative inputs from instructional designers introduces potential biases. The quality of outputs in the Skills and Profile modules depends on the accuracy and expertise of the human actors who complete them. This subjectivity, while inherent to educational design, raises questions about the consistency of applications across contexts.

Third, the integration of AI in ATHENA is at an early stage. While generative models assist in mapping competencies to pedagogical activities, the absence of adaptive learning loops limits the system’s ability to provide personalized, data-driven recommendations over time. Incorporating robust learning analytics while ensuring ethical safeguards for data privacy will be a critical step in future development.

Finally, the model’s complexity may pose barriers to adoption. With sixty facets and four levels, ATHENA requires substantial familiarization before it can be effectively used by practitioners. Simplified interfaces, training for instructional designers, and iterative feedback loops will be essential for large-scale diffusion.

### 7.5. Future Directions

Future research should address these limitations by pursuing three avenues. First, empirical validation of the ATHENA ontology through psychometric studies, experimental designs, and cross-professional comparisons will be needed to assess its reliability and predictive validity. Second, the development of adaptive, AI-driven functionalities, while maintaining human oversight, can enhance the responsiveness of the system to learner progress and contextual dynamics. Third, cross-cultural studies will be valuable to examine whether the ATHENA structure, though anchored in European frameworks such as ESCO, is applicable in diverse sociocultural and professional environments.

## 8. Conclusions

The ATHENA competency model offers a novel contribution to the psychology of creativity and skill development. By integrating cognitive, conative, knowledge-based, emotional, and sensorimotor dimensions into a systemic and operational framework, it provides a new lens for understanding and fostering creative performance in professional contexts. While challenges remain in terms of empirical validation and practical adoption, ATHENA lays the foundation for a research–practice interface that bridges psychological theory, educational design, and technological innovation. In this sense, it contributes not only to the science of creativity but also to the broader project of equipping individuals and organizations to navigate the complex challenges of the 21st century.

## Figures and Tables

**Table 1:** Comparison between known frameworks and ATHENA.

Framework	Conceptual Focus	Structure	Primary Use	Distinctive Features
KSAOs (Fleishman & Reilly, 1992)	Individual attributes (knowledge, skills, abilities, other characteristics)	4 categories	Job analysis and selection	Static trait taxonomy; lacks developmental progression.
O*NET (U.S. Dept. of Labor)	Occupational descriptors and skills	Hierarchical database	Labour-market mapping	Descriptive; no systemic linkage between cognitive, emotional, or motivational facets.
Four-C Model (Kaufman & Beghetto, 2009)	Levels of creative expression (Mini-C → Big-C)	4 levels	Creativity research and education	Focus on creativity, not general competency; lacks operational facets.
ESCO (European Commission, 2023)	Taxonomy of skills and occupations	Multi-level classification	Workforce standardization	Comprehensive but non-developmental;
ATHENA Model (Lamri & Lubart, 2023)	Agentic coordination of cognitive, conative, emotional, knowledge, and sensorimotor resources	5 dimensions × 12 sub-dimensions × 60 facets × 4 levels	Research, instructional design, AI-assisted learning	Dynamic, systemic, developmental, and integrable with digital learning platforms. ATHENA builds semantic interoperability with ESCO.

**Table 2:** Overall ATHENA Learn objectives for each ATHENA dimension.

Dimension ATHENA	Priority Educational Objective	Action Requested from the Learner During the Training Action	Observable Behavior During the Training Action
Cognition	Develop the ability to actively process information to analyze, reason, and solve complex problems.	Mobilization of attention, reasoning, abstraction, evaluation, metacognition.	Production of arguments, problem solving, diagramming, analytical discussion.
Knowledge	Enable the learner to acquire, organize and mobilize explicit knowledge, facts, concepts or theories.	Encoding, mental structuring, activation of concept networks, categorization.	Memorization, application of rules, synthesis of knowledge, questioning of facts.
Conation	Stimulate the intention to act, motivation, perseverance and the ability to set and pursue goals.	Motivational activation, intentionality, projection into action, effort management.	Taking initiative, verbalizing intention, active engagement in the task.
Emotion	Help identify, understand, express and regulate emotions in learning or social contexts.	Emotional activation, recognition of internal signals, affective regulation.	Verbal expression of emotions, empathic posture, behavioral adjustment to others.
Sensorimotion	Develop perceptual and motor coordination in interaction with the environment or physical objects.	Sensory-motor coordination, postural adjustment, spatial or gestural processing.	Object manipulation, motor simulation, physical response to a sensory stimulus.

**Table 3:** ATHENA Learn characteristics by learning level.

Level	Type of Learning	Guidance in Learning	Complexity of the Subject/Task to Be Carried out in Learning	Autonomy Expected from the Learner
1—Beginner	Simple task, strongly guided	Pupil	Weak	Weak
2—Intermediate	Semi-guided task, discovery of strategies	Moderate	Average	Average
3—Advanced	Open task, combined with several dimensions	Weak	High	High
4—Expert	Complex, self-regulated, reflective task	Very weak	Very high	Very high

**Table 4:** Pedagogical activities to develop divergent thinking, organized by expertise level.

**Level 1: Generates a few simple ideas, mainly based on existing or obvious solutions.**	**Guided exploration of simple variations**	**List of constrained ideas**	**Free association**	**Visual narrative triggers**	
	**Description:**	**Description:**	**Description:**	**Description:**	
	The learner explores several variations based on a basic element (e.g., sentence, object, mini-scenario), with simple instructions to encourage the production of ideas without judgment.	The learner generates ideas while respecting a simple constraint (e.g., not using a word, limiting oneself to a category). This facilitates creative emergence through slight restriction.	The learner makes associations of ideas based on triggering stimuli (images, words), while maintaining an imposed theme. Encourages controlled deviation and surprise.	The learner starts with an unexpected or unusual image and invents several interpretations. This allows for the initiation of divergent thinking through visual disruption.	
	**Example of synchronous activity:**	**Example of synchronous activity:**	**Example of synchronous activity:**	**Example of synchronous activity:**	
	In the classroom: Using an everyday object, learners must imagine three new possible uses. The trainer guides them with structured prompts.	Workshop: Each group must invent five ways to improve a service without using money. An oral presentation will be organized.	In the classroom: Each learner draws two random words + a theme (e.g., “trust”). They must create a mini-story linking the two words to the theme.	In the room: projection of an ambiguous photo. Participants note three different hypotheses about the scene, then discuss them.	
	**Example of asynchronous activity:**	**Example of asynchronous activity:**	**Example of asynchronous activity:**	**Example of asynchronous activity:**	
	On the platform: a micro-scenario is presented; the learner suggests 3 different options via a form, with automatic feedback encouraging diversity.	Exercise: The participant completes a form where they must generate 5 name ideas for a product… without using certain forbidden words.	Online: Random word generator with instructions to create a product idea linked to a given customer need.	On platform: presentation of a visual + instruction to imagine 3 press article titles describing this scene from different angles.	
**Level 2: Produces several varied ideas, beginning to explore less conventional approaches.**	**Brainwriting**	**Divergent heuristic mapping**	**Analog Association**	**SCAMPER**	
	**Description:**	**Description:**	**Description:**	**Description:**	
	The learner generates several ideas in writing while following a light constraint (e.g., imposed theme, type of innovation). Allows for increased fluidity and flexibility in creative production.	Creation of an exploratory mind map where the learner must multiply the divergent branches from a central theme, looking for unexpected or creative links.	The learner draws creative analogies between a given problem and distant domains, using a corpus of trigger examples to stimulate the gaps.	Application of the SCAMPER method (Substitute, Combine, Adapt, etc.) on an existing product or service, with guiding questions to stimulate structured variations.	
	**Example of synchronous activity:**	**Example of synchronous activity:**	**Example of synchronous activity:**	**Example of synchronous activity:**	
	In the classroom: Team exercise. Each student writes three ideas on a sheet of paper, then passes it to their neighbor to respond to. Several rounds are completed before sharing.	In the classroom: collective creation of a giant map on a whiteboard around a challenge (“how to reinvent urban transport?”).	In the classroom: based on a business problem, participants randomly draw images or concepts from another sector (nature, art, sport) and must find an inspirational link.	In the classroom: group animation of a SCAMPER on an everyday object with subgroups each working on two different letters.	
	**Example of asynchronous activity:**	**Example of asynchronous activity:**	**Example of asynchronous activity:**	**Example of asynchronous activity:**	
	Online: collaborative platform where participants submit 3 initial ideas, then enrich 2 ideas from other participants with structured comments.	On platform: digital mind map where the learner adds at least 10 different ramifications, with encouragement to explore several unexpected axes.	On platform: guided exercise where the learner receives 3 unusual images and must extract possible solutions for an imposed challenge.	Online: Interactive SCAMPER module where the learner answers guided questions to modify an existing product or service.	
**Level 3: Generates many original and diverse ideas, actively exploring unique perspectives.**	**Cross-Brainstorming**	**Reflection matrices**	**Reverse analogies**	**Random stimuli**	**Recombination Challenge**
	**Description:**	**Description:**	**Description:**	**Description:**	**Description:**
	Two groups brainstorm separately on the same topic, then exchange ideas to enrich, recombine, or transform the initial proposals. Stimulates advanced creative flexibility.	Create a matrix crossing two dimensions (e.g., user needs x emerging technologies) to generate ideas at the intersection of the axes. Requires multidimensional thinking.	Use of reverse analogies: Instead of looking for similarity, the learner looks for opposites or counterexamples to stimulate divergence.	Use of offbeat stimuli (absurd images, surreal sentences) to provoke cognitive deviations, followed by an in-depth analysis of possible links with the initial challenge.	Using existing ideas (from previous exercises), the learner must create a hybrid solution combining several ideas, promoting originality.
	**Example of synchronous activity:**	**Example of synchronous activity:**	**Example of synchronous activity:**	**Example of synchronous activity:**	**Example of synchronous activity:**
	In the classroom: two teams work separately for 15 min, then cross-reference their lists and must create 5 new ideas based on the other group’s ideas.	In the classroom: collective creation of a matrix on a board (axes chosen together), then systematic search for ideas at each intersection.	In the classroom: exercise where each learner must, from a concept (e.g.,: collaboration), imagine its opposite (e.g.,: isolation) and extract creative ideas.	In the classroom: the trainer displays 5 absurd images, each group must connect them to the challenge to be solved by logically justifying the links.	In the classroom: After a classic brainstorming session, each participant chooses two different ideas from another group and creates a hybrid proposal.
	**Example of asynchronous activity:**	**Example of asynchronous activity:**	**Example of asynchronous activity:**	**Example of asynchronous activity:**	**Example of asynchronous activity:**
	On the platform: each group submits its list of ideas; the other group proposes improvements or recombinations on a dedicated forum.	Online: interactive digital matrix where the learner must propose 2 ideas per combination of imposed axes.	On platform: random drawing of an inverted concept on an interface, then creation of a solution derived by contrast.	On platform: bank of absurd stimuli; the learner draws 2 stimuli and proposes a project which combines their characteristics.	On platform: exercise of recombination of ideas collected during previous phases of a participatory innovation project.
**Level 4: Creates a constant flow of highly innovative and adaptive ideas, pushing the boundaries of conventional thinking.**	**Design fiction**	**Provocative narrative divergence**	**Conceptual hybridization**	**Cognitive disruption strategy**	**Biomimicry**
	**Description:**	**Description:**	**Description:**	**Description:**	**Description:**
	Creation of a complex speculative scenario that explores the societal, technical, and human impacts of a radical innovation. Mobilizes systems thinking and critical imagination.	Voluntary exploration of absurd, taboo, or extreme scenarios to stimulate creative change and cognitive liberation. Allows you to break out of traditional thought patterns.	The learner combines several distant concepts from distinct fields to generate original innovations. The activity is based on unconventional recombinations.	A method of questioning all the assumptions of a given system or product using techniques of provocation or reversal of implicit postulates.	An innovation method inspired by living systems to create sustainable and original solutions. It stimulates divergent thinking by shifting the focus from human to living systems, thus encouraging the emergence of new ideas through deep analogy.
	**Example of synchronous activity:**	**Example of synchronous activity:**	**Example of synchronous activity:**	**Example of synchronous activity:**	**Example of synchronous activity:**
	In the room: co-design fiction workshop where each group imagines a plausible future (20 years from now) based on an emerging technology, then scripts its impact on several levels (society, work, environment).	In the classroom: Based on a serious topic (e.g., education), participants must create provocative, absurd, or dystopian stories, then extract a useful lesson or unexpected innovation from them.	In the room: each group draws two very different concepts (e.g., circular economy + immersive theater) and must design a conceptual prototype.	In the classroom: Each group identifies five implicit assumptions of a product/service. They must then propose an innovative version in which each of these assumptions is reversed.	In the classroom: Each group analyzes a challenge (e.g., reducing friction, producing without waste) by observing biological mechanisms (the structure of the lotus, shark skin, etc.). They translate these observations into concepts applicable in their own context.
	**Example of asynchronous activity:**	**Example of asynchronous activity:**	**Example of asynchronous activity:**	**Example of asynchronous activity:**	**Example of asynchronous activity:**
	On platform: Creation of an illustrated fictional story presenting a world transformed by an innovation. The learner receives peer feedback on coherence, originality, and critical value.	On platform: Divergent writing exercise with absurd constraint generator. The learner must justify how the story opens up unexpected avenues of innovation.	On platform: digital conceptual hybridization tool with library of concepts to cross-reference and produce an innovative idea pitch.	On platform: guided disruption module: the learner chooses a system, identifies its implicit rules, and proposes a radical alternative for each.	On platform: guided workshop with CK digital mapping tool. The learner must formulate an original concept, then explore its development by gradually integrating blocks of knowledge (scientific, technical, societal).

## Data Availability

The original contributions presented in this study are included in the article. Further inquiries can be directed to the corresponding author.
